# Chief nursing officers’ perspectives on Medicare’s hospital-acquired conditions non-payment policy: implications for policy design and implementation

**DOI:** 10.1186/1748-5908-7-78

**Published:** 2012-08-28

**Authors:** Heidi Wald, Angela Richard, Victoria Vaughan Dickson, Elizabeth Capezuti

**Affiliations:** 1 School of Medicine, University of Colorado Denver, Aurora, 80045, CO, USA; 2 College of Nursing, New York University, New York, 10003, NY, USA

**Keywords:** Quality improvement, Health policy, Patient safety

## Abstract

**Background:**

Preventable adverse events from hospital care are a common patient safety problem, often resulting in medical complications and additional costs. In 2008, Center for Medicare and Medicaid Services (CMS) implemented a policy, mandated by the Deficit Reduction Act of 2005, targeting a list of these ‘reasonably’ preventable hospital-acquired conditions (HACs) for reduced reimbursement. Extensive debate ensued about the potential adverse effects of the policy, but there was little discussion of its impact on hospitals’ quality improvement (QI) activities. This study’s goals were to understand organizational responses to the HAC policy, including internal and external influences that moderated the success or failure of QI efforts.

**Methods:**

We employed a qualitative descriptive design. Representatives from 14 Nurses Improving Care of Health System Elders (NICHE) hospitals participated in semi-structured interviews addressing the impact of the HAC policy generally, and for two indicator conditions: central-line associated bloodstream infection (CLABSI) and catheter-associated urinary tract infection (CAUTI). Within-case analysis identified the key components of each institution’s response to the policy; across-case analysis identified themes. Exemplar cases were used to explicate findings.

**Results:**

Interviewees reported that the HAC policy is one of many internal and external factors motivating hospitals to address HACs. They agreed the policy focused attention on prevention of HACs that had previously received fewer dedicated resources. The impact of the policy on prevention activities, barriers, and facilitators was condition-specific. CLABSI efforts were in place prior to the policy, whereas CAUTI efforts were less mature. Nearly all respondents noted that pressure ulcer detection and documentation became a larger focus stemming from the policy change. A major challenge was the determination of which conditions were ‘hospital-acquired.’ One opportunity arising from the policy has been the focus on nursing leadership in patient safety efforts.

**Conclusions:**

While the CMS’s HAC policy was just one of many factors influencing QI efforts, it may have served the important role of drawing attention and resources to the targeted conditions—particularly those not previously in the spotlight. The translational research paradigm is helpful in the interpretation of the findings, illustrating how the policy can advance prevention efforts for HACs at earlier phases of research translation as well as pitfalls associated with earlier phase implementation. To maximize their impact, such policies should consider condition-specific contextual factors influencing policy uptake and provide condition-specific implementation support.

## Background

As a group, preventable adverse events related to hospital care are a common, morbid, and costly patient safety problem [1]. Until 2008, the Center for Medicare and Medicaid Services’ (CMS) Medical Severity-Diagnosis Related Group (MS-DRG) system was unable to distinguish preventable adverse events from comorbid conditions. This limitation had the effect of providing a perverse financial incentive to hospitals based on the Inpatient Prospective Payment System (IPPS). Under this regime, hospitals were eligible for increased reimbursement for the care of patients who suffered preventable adverse events in the hospital [2].

Beginning on 1 October 2008, hospitals received a new financial incentive to improve practice in a manner aligned with patient safety priorities selected by Medicare in conjunction with the Centers for Disease Control and Prevention (CDC) [3]. This rule change, mandated by the Deficit Reduction Act of 2005, targeted a select list of eight ‘reasonably’ preventable adverse events referred to as hospital-acquired conditions (HACs) for reduced reimbursement. In 2009, this list was expanded to 10 conditions [4].

The HAC policy was the subject of highly publicized debate among consumers and providers of care [2,5]. Concerns were raised about potential adverse moral, technical, and safety implications of this performance-sensitive payment method. Specifically, given that few of the HACs are uniformly preventable, some felt it was unjust to penalize providers when there were likely to be instances of unavoidable harm even if exemplary care was provided [6]. Others argued that the use of billing data to identify HACs was inaccurate [7]. Finally, others warned of the unintended consequences of the focus on specific conditions for patient outcomes and for providers who might face increased burden and scrutiny. For example, an unyielding focus on fall prevention might unintentionally result in increased use of restraints, decreased mobility, and functional decline for vulnerable patients [8]. There was little mention, however, of the potential for a positive impact of the HAC policy on the implementation of evidence-based prevention strategies through hospitals’ quality improvement (QI) activities [2].

In formulating a research agenda for understanding the impact of CMS’s HAC policy on hospital-acquired infections (HAI), Stone, et al. [9] presented a conceptual model in which both the incentive (the HAC policy) and other environmental factors (payment, regulatory, and market factors) act upon organizational factors (leader behavior, organizational culture, and staff behavior) to produce desired outcomes (clinical, staff, financial). The primary goal of this qualitative descriptive study was to explore hospitals’ organizational responses to the HAC policy—primarily QI activities—and the organizational and environmental influences that moderated the success or failure of these efforts. We hypothesized that prevention activities for each type of HAC pose unique implementation issues and therefore, the impact of Medicare’s HAC policy on hospitals’ QI activities would differ depending on the HAC examined.

### Indicator HAC’s: CAUTI and CLABSI

To explore this hypothesis, we selected two indicator HACs: central line-associated bloodstream infection (CLABSI) and catheter-associated urinary tract infection (CAUTI). Despite the many similarities in prevention strategies for these device-associated infections, CLABSI prevention occurred in a very different context from that of CAUTI prevention in 2007 and 2008. The translational research paradigm is useful for describing these contextual differences. Five phases of translational research have been identified (adapted from Institute of Translational Health Sciences https://www.iths.org/about/translational):

• T0. Identify problems, opportunities, and approaches

• T1. Discovery or foundational research

• T2. Health application to assess efficacy

• T3. Health practice; science of dissemination and implementation

• T4. Evaluation of health impact on real world populations

Applying this paradigm, CLABSI prevention might have been characterized as phase T3 at the time of the rollout of the HAC policy with: national voluntary QI efforts focused of CLABSI (i.e., Institute for Healthcare Improvement [IHI]); well-publicized, large-scale successes in CLABSI prevention [10,11]; and a federally-funded implementation effort which was by 2009 expanded to all 50 states. In contrast, CAUTI prevention might have been characterized as phase T2 at the time of the HAC policy rollout. Not only did CAUTI prevention lack parallel efforts to CLABSI, but both the CDC’s evidence-based guidelines for CAUTI prevention and the revised CAUTI surveillance definition were released after the HAC policy was in effect [12,13]. Therefore organizational (*e.g.*, surveillance) and environmental (*e.g.*, QI collaboratives) efforts to support CAUTI prevention lagged behind those for CLABSI prevention. The contrast between the context for CLABSI and CAUTI prevention activities presents an opportunity to explore the impact of the HAC policy on each condition and to inform the successful design of future initiatives.

## Methods

Nurses Improving the Care of Health System Elders (NICHE) is a national geriatric nursing program providing clinical, educational, and organizational resources to member-hospitals and their affiliates to support excellence in hospital care of older adult patients (http://nicheprogram.org/). For this study, NICHE hospitals represented a convenience sample of acute care hospitals that may be atypical in several respects when compared with all US hospitals. First, NICHE hospitals may have a higher level of commitment to and investment in nursing practice both regard to geriatric nursing specifically and to nursing practice more generally, as evidenced by the large proportion of NICHE hospitals that have achieved Magnet designation from the American Nurses Credentialing Center of the American Nursing Association (32% of NICHE hospitals versus <8% of all US hospitals http://www.nursecredentialing.org/). This orientation most certainly influenced their interest in addressing the geriatric, nursing-sensitive conditions that appear on the HAC list (pressure ulcers, falls with injury, and catheter-associated urinary tract infection). Second, NICHE hospitals are overwhelmingly not-for-profit institutions.

We conducted a descriptive qualitative study with NICHE Chief Nursing Officers (CNOs). Given the limited scope of the project, it was necessary to narrow the focus to a single hospital representative. We selected the hospital CNO because of her broad perspective on frontline practice, the challenges faced by frontline staff, QI initiatives, hospital organizational structures and incentives, and local and national health policy.

The study was exempted from oversight by the Colorado Multiple Institution Review Board (COMIRB) because it was not considered human subjects research. A list of NICHE hospitals was obtained from the NICHE central office at New York University and stratified based on whether state reporting of HAIs was required at the time of the HAC policy implementation [14]. This stratification was carried out because state-mandated reporting of HAIs is an environmental factor that in 2008 was applied non-randomly to hospitals in 24 states. Public reporting is considered a reputational rather than financial incentive to hospitals, and therefore could reasonably be expected to have an independent impact on hospitals organizational responses to HAI prevention [9]. A random list of 60 hospitals—30 for each group—was generated. Each CNO was sent a recruitment letter explaining the project and requesting indications of interest. Within two weeks of the mailing, CNOs were re-contacted via email, phone, and/or fax, with up to two additional email or phone contacts for non-responders. Two hospitals, one from each stratum, were screened out because they had unfilled CNO positions. Fifty-eight hospitals were contacted and fourteen (23%) agreed to participate, seven from each stratum. Site characteristics were obtained from the NICHE Benchmarking Service that includes institutional data from both a biennial Self-Evaluation survey and the annual American Hospital Association database.

Two researchers trained in qualitative interviewing techniques conducted telephone interviews with CNOs, guided by a semi-structured interview guide to elicit in-depth narratives about their the overall impact of the HAC policy and the hospital’s response to the policy, specifically considering CAUTI and CLABSI. The interview guide questions can be grouped under each of the primary model domains from the Stone framework. After obtaining verbal permission for the interview and tape recording, the interview began with a series of open-ended questions followed by focused probes. The first questions focused on overall hospital organization and the role of staff at various levels in QI efforts (*e.g.*, ‘how engaged is your Board of Directors in quality improvement efforts?’). Probes were used to explore specific areas of interest among organizational-managerial influences. Next, questions focused on environmental drivers of QI priorities, both market factors (*e.g.*, participation in collaboratives and benchmarking programs) and state regulations (*e.g.*, state-mandated reporting of HACs, if present, and the influence of the Medicare HAC non-payment policy). Interviewees were asked to fully describe the specific efforts implemented at their institution to reduce CAUTI and CLABSI and organizational factors influencing the success of these efforts. They were asked for examples and to clarify any unclear descriptions. The researcher used phrases such as ‘what do you mean by that?’, ‘explain that to me again,’ or ‘I don’t follow you exactly’ to clarify remarks rather than assume what the participant meant.

Consistent with qualitative research methodology, we used purposive sampling to select a small number of individuals who were able to provide in-depth information about the research question, rather than a large group of individuals. Our interviews continued until data saturation occurred (i.e. no new themes were revealed by additional interviews), which was evident after identifying recurring themes in the qualitative analysis [15]. A total of 16 interviews were conducted; 13 with CNOs and three with other hospital staff (one QI officer, one CEO, and one nursing quality program manager). Data saturation was achieved at interview 16, which is a fairly typical sample size for qualitative descriptive studies. [16] All interviews were conducted between May and September 2010.

The recorded interviews were transcribed. Observational data including interview length and any deviations from the interview protocol were collected using field notes and entered into Atlas.ti to augment the analysis. Due to an audiotape recorder malfunction, two interviews were not taped. Extensive notes were used to reconstruct those two interviews, and the notes reviewed by the participants for accuracy. To ensure consistency in the interview process, the first five interviews were conducted by both the PI and co-investigator. Each tape-recorded interview was reviewed as to fidelity in the use of the interview guide, tone of interview, and handling of respondent questions. When schedules permitted, interviews were conducted jointly. In total, 10 interviews were conducted by both investigators; six were conducted by a single investigator.

### Data analysis

Following the guidelines set by Sandelowski [17], qualitative data analysis began immediately once the taped interviews were transcribed. Data were transcribed verbatim and transcripts checked for accuracy. The transcribed data were analyzed using content analysis, an inductive analytic method, to identify institutional response to the HAC policy, and barriers and facilitators to the implementation of QI initiatives. Atlas.ti software (version 6.2) was used in this analysis. Preliminary coding was based on a list of *a priori* codes generated by the project team, with additional codes identified during the coding of the first few interviews. The initial coding scheme resulted in categories of data related to research questions on institutional response to the HAI policy and influences including facilitators and barriers.

Using a case-oriented approach, each of the hospitals constituted a case. After preliminary coding, in-depth within-case analysis was used to identify the key components of each institution’s response [18]. Subsequently, across-case analysis focused on the categories that recurred across the 14 cases in order to identify commonalities and variations of the themes across the entire sample [19]. Themes emerged from the categories to identify how the hospitals responded to the HAC policy and the influences on those responses, the focus of the research questions. To do this, an informational matrix was developed to analyze the themes across the fourteen hospitals with consideration to characteristics, such as state reporting requirements, size, rural/urban location, and teaching/nonteaching status. The matrix was reviewed to identify any qualitative differences in hospital experiences potentially related to these characteristics. The analysis process was iterative, whereby the researchers moved back and forth between individual cases and across cases to track variability. Ongoing project team discussions allowed for the review of emerging themes. Finally, exemplar cases were identified to explicate the findings.

Attention to methodological rigor was given through all phases of the analysis. As noted above, all interview transcriptions were reviewed verbatim for accuracy. The first two transcribed interviews were coded independently by two coders and the coding was reviewed for similarities and variations. Initial discrepancies were discussed and resolved. The proportion of coded items where both coders agreed on the coding was assessed. Ninety-five percent agreement was achieved suggesting that the coding scheme used was appropriate [20].

## Results

Characteristics of the 14 participating hospitals appear in Table 1. Responding hospitals were compared to non-responding hospitals (n = 44) with respect to numbers of beds, urban/rural location, US region (northeast, south, Midwest, west), teaching/nonteaching, ownership, and magnet status. None of the comparisons were significantly significant (p < 0.10). During the interviews, we learned that one hospital had been misclassified as being in a non-reporting state. This hospital was moved to the reporting state group for the purposes of analysis. We conducted 16 interviews with a total of 19 participants. Thirteen interviews were with CNOs, two were with hospital executives referred by the CNO, and one was with a nursing quality program manager. Additional hospital staff participated on two calls at the CNO’s request. During these two interviews, the CNO elected to include clinical staff to provide additional detail on how QI efforts were affecting bedside care on hospital units. These interviewees were able to provide rich detail on staff response to QI efforts and the degree to which best practices were implemented. The content thematic analysis [17] revealed three overarching themes identifying the internal and external influences on institutions’ responses to the HAC policy and highlighting challenges and opportunities prompted by the policy.

**Table 1:** Characteristics of participating hospitals and interviewees (stratified by HAI reporting status)

**Characteristic**	**Reporting (n = 8)**	**Non-Reporting (n = 6)**
**Hospitals**		
Bedsize (mean, (range))	(346, (168 to 606))	(434, (201 to 750))
Urban	75%	100%
Geographic Location		
Northeast	88%	0%
South	0%	50%
Midwest	0%	50%
West	12%	0%
Teaching Hospital	88%	50%
Ownership		
Not-for-profit	100%	83%
Government	0%	17%
Magnet hospital	63%	0%
**Interviewees**		
Position	**n = 12**	**n = 7**
Chief Nursing Executive	67%	71%
Chief Operating Officer	25%	0%
Chief/Other Quality Officer	8%	0%
Other RN	0%	29%
Years in position (mean, (range))	(5.2, (4 Months to 22 Years))	(8.8, (1 Year to 20 Years))
Years at institution (mean, (range))	(4.7, (10 Months to 11 Years))	(11.1, (10 Months to 33 Years))
Terminal degree (%)		
PhD	25%	14%
Any 1 Master’s Degree (MSN, MBA, MNA, MHA, MEd)	50%	72%
MSN, MBA (combo)	17%	14%
MD	8%	0%

### Selection and timing of QI activities: internal, external, and condition-specific

Respondents identified many organizational and environmental influences on the timing and selection of QI activities (Table 2). Organizational influences most often cited were institutional experience, resources, and other organizational characteristics. Institutional experience refers to the perception of a quality or patient safety problem, often based on surveillance metrics (i.e., infection control reports of CLABSI rates). The influence of both material and human resources were discussed. Interviewees noted that scarcity of material resources (i.e., bladder scanners) and inability to collect data delayed initiation of CAUTI prevention activities. Important human resources included the presence of a quality ‘champion’ to drive QI activities (*e.g.*, a clinical nurse specialist interested in implementing a nurse-driven catheter removal protocol).

**Table 2:** Influences on timing and selection of hospital quality improvement activities

**Influence**	**Example**
**Internal**	
1. Experience	Surveillance of central line infection rates by unit
2. Resources	
a. Material	Lack of bladder scanners, lack of data collection infrastructure for measuring catheter-days
b. Human	Hospital epidemiologist with interest in central line infections
3. Organizational Characteristics	Performance improvement committee of governing board; part of multi-hospital system monitoring performance indicators
**External**	
1. Voluntary/professional	Michigan Keystone, NDNQI*
2. Regulatory	HAC** policy; CMSº core measures, Joint Commission
3. Financial	Non-federal pay for performance (Blue Cross Blue Shield)

*NDNQI = National Dataset of Nursing Quality Indicators.

**HAC = Hospital-acquired Conditions.

ºCMS = Centers for Medicare and Medicaid Services.

Organizational characteristics influencing QI efforts included being part of a hospital network (where some QI efforts were undertaken for the group of hospitals) and the degree of board engagement in QI programs. As one interviewee described:

"‘They [the board] are very engaged. We start each of our Board meetings with 30 minutes related to patient safety and quality…’"

Interviewees discussed multiple environmental influences on QI activities. Many participated voluntarily in national or regional collaboratives (*e.g.*, the IHI campaign, state hospital association collaboratives, et al.), professional organization benchmarking programs, or regional professional societies. These influences provided guidance on best practices and/or benchmarked quality indicator reports. Regulatory influences included public reporting (CMS’s Hospital Compare Website and state-mandated reporting of HAIs and HACs).

Interestingly, payment incentives including the HAC policy, pay for reporting programs, and other payment incentives driven by third-party payers (*e.g.*, Blue Cross Blue Shield) were identified as having influence on QI activities, but not serving as the primary driver of those activities. For example, one CNO stated:

"‘We were already doing some deep dives on this stuff before it became mandated. I think certainly there is some [effect of the HAC policy] because it reinforces for the finance people that we really do have to do this.’"

Most respondents indicated that the HAC policy had both illuminated existing activities and raised the profile of HACs that had previously received little attention and/or few resources. Several interviewees noted an increased awareness on the part of their hospital’s board about HACs and increased access to resources for QI activities. As one CNO explained:

"‘… You can get the resources allocated more easily at the organizational level…. but our concern about quality of care issues has always been high…we were already doing these things, it’s just raised awareness….’"

For two hospitals, however, the HAC policy did serve as a key driver for QI initiatives directed toward CAUTI reduction. One respondent stated:

"‘All [the QI efforts directed toward CAUTI prevention] came out because of it.’"

### Condition-specific responses

Interviewees from all 14 hospitals described CLABSI-directed activities, while 11 described CAUTI QI initiatives. Of the three with no pre-existing CAUTI initiative, one was planning a CAUTI QI project and another had initiated a project that hadn’t ‘gained traction.’ Respondents mentioned QI programs targeted toward other HACs including pressure ulcers (n = 11), falls (n = 5), and deep venous thrombosis (n = 2). Table 3 summarizes QI activities by condition including timing, focus, barriers, facilitators and exemplars.

**Table 3:** Condition-specific QI activities

**Condition**	**Timing**	**Focus**	**Barriers**	**Facilitators**	**Exemplars**
CLABSI	Pre-existing	Prevention	Attribution (Present on Admission), Implementation, Collaboration	Access to correct equipment, Self-policing, Collaboration with external groups	Access to correct equipment: ‘You have to do full garb, full layout of sterile field…we developed carts that have everything on it, so it made everybody’s life easier.’ Self-policing: ‘They also track central line infections… a unit that had a particular spike…did some special follow-up… they actually got right back on track.’ Collaboration with external groups: ‘…because everybody (in the state hospital association) was doing it…where you met with physician resistance, you could say, well, it’s being done at the hospital next door and the hospital north of us, south of us, east of us, west of us….’
CAUTI	Concurrent or planned	Prevention and Surveillance	Attribution (Present on Admission)	Adequate resources Piloting prior to scale up	Adequate resources: ‘(prior to the policy) we were sharing (bladder scanners) between several units. Well, that’s not good enough…if you’re looking to get to zero, you have to have it as part of their practice.’ Piloting prior to scale up:’… a phenomenal geriatric CNS…has worked with us to try to…reduce or prevent CAUTIs in the geriatric patients across the hospital…we ‘started’ on our geriatric ACE unit ahead of time to help us put a template in place.’
Pressure Ulcer	Concurrent	Screening and documentation	Attribution (Present on Admission), Shared responsibility, Preventability, Collaboration	Collaboration with physicians, External reporting, and benchmarking	Collaboration with physicians: ‘We knew our patients had them (pressure ulcers), but the doctors didn’t because it wasn’t on the forefront of what they do, and now that they have to document, they’re in there looking at the wound with the nurse…’ External reporting and benchmarking:’… we belong to NDNQI so we do the actual assessment of all patients quarterly and then do a rate…’

### CLABSI

Most CLABSI-directed activities described in the interviews were in place prior to the HAC policy rollout. The most commonly described approach was bundled prevention interventions (n = 8) that were either developed internally or adopted from an external organization such as IHI. The success of these initiatives was facilitated by the availability of adequate resources (*e.g.*, equipment packs) and institutional commitment through self-policing and/or surveillance methods, as well as collaboration with external entities such as IHI.

### CAUTI

Fewer hospitals had well-established QI initiatives addressing CAUTI. Several were still considering their approach or talked of plans in development. CAUTI interventions were more heterogeneous than those described for CLABSI. Most commonly mentioned were the use of nursing protocols for indwelling urinary catheter insertion, care, and/or removal and the establishment of tracking methods for CAUTIs and catheters.

Several interviewees admitted that the HAC policy played a significant role in focusing their institution’s attention on the issue of CAUTI. As one CNO stated:

"‘We did need to address it [CAUTI] but I don't know if we would have addressed it in this manner [without HAC Policy].’"

Frequently, CAUTI efforts were facilitated by piloting a program on one unit, and then expanding to other units. Resource availability including use of nursing specialists, bladder scanners, and surveillance practices also facilitated successful efforts.

### Pressure ulcer

While pressure ulcers were not one of the HACs selected for focus for this project, 11 interviewees raised the topic of the impact of the policy on hospital-acquired pressure ulcers. Most reported that their institutions had a preexisting clinical focus on pressure ulcers, but that the HAC policy had spurred increased attention to evaluation and documentation of pressure ulcer prevention, particularly at the time of hospital admission. Collaboration with physicians and external reporting of pressure ulcer prevalence to programs such as the American Nursing Association’s National Database of Nursing Quality Indicators (NDNQI) [21] were both cited as facilitators for pressure ulcer initiatives.

### Challenges arising from the HAC policy: attribution and prevention

Although many examples were cited of successful efforts to decrease HACs, interviewees identified two primary challenges: attribution and prevention. The challenges differed depending on which HAC was being discussed (Table 3).

### Challenge of attribution

For the purposes of this discussion, the term ‘attribution’ refers to the identification of the site of care where the patient acquired a medical condition. Attribution is key to the determination of whether an HAC was present on admission (POA) or hospital-acquired. While attribution was noted to be a challenge for CLABSI and CAUTI, it was particularly relevant to pressure ulcer prevention. Many interviewees expressed concern that they were missing POA conditions due to lack of uniform screening or because conditions were pre-clinical. One described attempts to identify CLABSIs on patients with long-term central venous access:

"‘…It hadn’t been our practice to get cultures on those patients, so we think we’re taking some hits on infections that may have been there.’"

Another interviewee noted the challenges of identification of POA pressure ulcers:

"‘Of course, we don’t get the ones [pressure ulcers] we don’t know about, the ones that you’d have to have an ultrasound for.’"

There appeared to be two approaches to addressing this challenge. In lieu of universal screening for preclinical CAUTIs and CLABSIs, respondents reported increasing infection control surveillance activities. For pressure ulcer attribution, efforts focused on improving screening and documentation.

The second challenge related to attribution was shared responsibility for identification and documentation of POA conditions between nurses and physicians. This issue was also raised for pressure ulcers because skin assessment and care typically fall within the scope of nursing practice but regulatory requirements mandate physician documentation. Although some interviewees reported good success with collaborative efforts to document POA conditions, others experienced frustration:

"‘The tricky part is the doctor actually needs to document that part….So it's in flux here with how many doctors are actually doing it and how many are still going in kicking and screaming.’"

### Challenge of prevention

Challenges to implementing prevention activities included organizational barriers and lack of collaboration. Frustration was evident as CNOs described attempts to make progress with CAUTI prevention:

"‘We’re not measuring it [CAUTI], I think, as thoroughly as we need to, and we don’t understand our performance as well as we need to, and we don’t yet have the right champion and we don’t have the right team yet to really make the changes that we need to make there.’"

A prevailing sentiment was that development and adoption of nursing-driven catheter protocols and support of nursing autonomy were important strategies for CAUTI prevention. Nurse-physician collaboration again was described as critically important. Although numerous examples were given of successful collaboration (‘good support,’ ‘very collaborative,’ ‘major help…’), lack of collaboration was perceived as stalling important efforts:

"‘This [nursing-driven policy] has been to our executive committee four times… it is still meeting resistance from the physicians.’"

Another prevention challenge was the acknowledgment that some HACs may not be preventable despite exceptional nursing and medical care. This concept was repeatedly mentioned with regard to pressure ulcer preventability. Particularly salient was the example of the critically ill or severely compromised patient:

"‘…In an ICU, the primary motivating factor…is to save that person’s life…it really doesn’t lend itself to perfect skin condition.’"

Another interviewee articulated:

"‘How do you handle the patient with the unavoidable pressure ulcer…the patient at the end of life with terminal skin failure where nutrition can't be supported, where there are underlying disease processes causing their body to break down…?’"

While the ambiguity of ‘preventability’ was acceptable for some respondents, it proved a burden for others who expressed frustration at bearing the burden of responsibility for HACs and perceived failures in emotional terms:

"‘Does it look like the hospital’s fault? Sure…and that’s the damning piece about it.’"

### Opportunities arising from the HAC Policy: central role of nursing

The most commonly cited opportunity to arise from the HAC policy was in highlighting the roles of nurses as leaders, clinical innovators, and ‘guardians.’ Leadership roles included directing QI committees, functioning as liaison to executive committees, developing and advocating for resources and policies, and translating between key leaders and care providers. Nurses who were clinical innovators were described as developing and championing QI initiatives. The nursing ‘guardian’ or oversight role took the form of directing surveillance activities, self-policing for HAC events, leading quality activities such as root cause analysis, and group accountability.

Notably, interviewees expressed optimism for the field of nursing in general given a perception of an increased recognition of nursing’s role in patient safety afforded by the policy:

"‘This is the best thing that could happen to put the spotlight on the huge impact that nursing has…on patient outcomes. You’re that protector on the front line of the patient safety and quality of care.’"

There was also an underlying tone of pragmatism as CNOs described a balance between rising to the challenge and acknowledging the limits of prevention:

"‘When you think about what the percentage of non-preventable really are, I think the majority are preventable…it just takes a lot of intense energy and thought and care…’"

### Role of organizational characteristics

As noted previously, a matrix was developed to explore possible themes that varied according to hospital characteristics. Little variability in the themes that emerged in this data by organizational characteristics was found. While the influence of the institutional response to the HAC in the qualitative data was linked to organizational support, nearly all hospitals had a quality committee of the governing body and the responses did not allow for further discrimination of the other key features of that support. Internal barriers such as physician documentation or use of electronic health records did not appear to be variable by state reporting mandate.

## Discussion

Nearly two years after the implementation of Medicare’s HACs policy, NICHE hospital representatives reported several encouraging outcomes of the HAC policy, including raised awareness of HACs by hospital governing bodies. This translated to increased attention and resources for QI activities addressing HACs. Interviewees reported a variety of activities to enhance screening, documentation, collaboration, and surveillance for HACs since the 2008 rollout of the HAC policy. Finally, interviewees highlighted the opportunities for nursing professionals to demonstrate their ownership of HAC prevention and leadership in patient safety. Interviewees reported some discouraging outcomes of the HAC policy as well. In particular, frontline staff encountered significant difficulties in ensuring accurate attribution of HACs to the correct site of care. In addition, there was clear frustration with the limits of prevention activities. Although many environmental and organizational drivers of QI activities were identified, we were unable to discern important relationships between these drivers and the CNOs responses. In particular, we did not discern an effect of state-mandated HAI reporting on HAC-related QI activities, a finding which deserves additional exploration.

The selection of indicator conditions allowed us to explore condition-specific contextual considerations that may have enabled organizational responses to the HAC policy. Notably, interviewee responses suggest a differential impact of the HAC policy for each targeted condition, leading to important lessons for condition selection, policy implementation, and evaluation. While we specifically targeted CAUTI and CLABSI, the spontaneous emergence of comments about pressure ulcers during study interviews was remarkable and generally related to the dual challenges of attribution and prevention. Pressure ulcer prevention rests squarely in the domain of nursing care. There is a professional organization of nurses with extensive training in skin care, an array of prevention technologies, standardized surveillance definitions, an established methodology for determining prevalence, and large benchmarking projects, including the American Nurses Association National Database of Nursing Quality Indicators, implemented in the early 1990s [21], and the California Nursing Outcomes Coalition, beginning in 1996 [22,23]. Thus, pressure ulcer prevention might be characterized as at least T3 to T4 at the time of the HAC policy roll out.

We found the use of the translational research paradigm to describe the context for implementation of prevention activities for specific HACs to be helpful. Given that CAUTI prevention was at an earlier phase of research translation at the time of its selection to the HAC list, it is not surprising that CAUTI prevention activities were reported to be less developed and receiving fewer resources at the time of the HAC policy rollout. As a result, the selection of CAUTI to the HAC list might have been expected to have a disproportionately large impact on CAUTI prevention activities, relative to CLABSI and pressure ulcer. Certainly, respondents reported forging ahead in several cases with new CAUTI prevention activities, even though they were found to be challenging.

Selection of CAUTI to the HAC list may have accelerated implementation efforts, but caused other problems at the same time, because less is known about optimal prevention strategies and their dissemination. In particular, a poor understanding of how to implement CAUTI prevention practices may have lead to ill-considered approaches to CAUTI prevention and the expenditure of precious resources without attendant improvements in performance. Therefore the translational research context for a candidate HAC should be carefully considered prior to selection. For HACs in earlier stages of translation, accelerated and expanded rollout of supports for prevention activities should be prioritized.

One would expect trends in HAC outcomes would also reflect the sophistication of translational research efforts. For CAUTI, an increase in prevention activities might reasonably be expected initially, followed only later by a decrease in rates. For CLABSI, one would hope to see decreases in rates that closely track implementation of the HAC policy. For pressure ulcer, there is some evidence that pressure ulcer rates are beginning to plateau [24] perhaps explaining the concerns of respondents that the remaining hospital-acquired pressure ulcers may be unavoidable. Therefore, measures of success might be broadened account for these differences across conditions.

Several additional lessons can be derived from the challenges identified by our respondents including that cooperative work among providers was required to be successful in differentiating POA conditions from HACs and in performing prevention activities. There is increasing recognition that teamwork, as a component of a strong patient safety culture, is central to the success of HAI prevention initiatives [25]. Nonetheless, the focus on attribution of each HAC to a particular site of care does appear to pit providers against one another, especially across hospital units and post-acute care facilities. The design and implementation of future value-based purchasing initiatives should continue to seek ways to reward collaboration of providers within and across facilities.

Second, our respondents’ comments suggested that much of the challenge of attribution arising from the HAC policy is an artifact of the reliance on ICD-9 coding for HAC detection. The primary example from this sample arose in the context of pressure ulcer attribution. The process of documenting medical care highlighted a substantial procedural disconnect between nurses who have the clinical expertise and physicians who bear the medical record documentation burden. Other authors have noted that the complexity of billing for CAUTI is so cumbersome that few CAUTIs are identified by this methodology and the potential financial impact of the HAC policy minimized [26,27]. Because billing and surveillance are two different functions, it seems appropriate and important that they be decoupled. Surveillance activities have been standardized for CAUTI, CLABSI, and pressure ulcer [13,21]; however, they are not universally applied, and they do not exist for all HACs. The recent move to require CLABSI data collection to go through the CDC’s National Healthcare Safety Network is a step forward in this regard [28] and suggests that surveillance might one day take the place of billing in the determination of the HAC penalty.

Finally, the nursing executives interviewed actively embraced the challenges presented by the HAC policy and described leadership roles in administrative, clinical, and oversight roles related to patient safety activities. These roles need to be adequately supported with specialized training, resources, and professional stature. An emphasis on the crucial role of nursing in patient safety should resonate with policymakers struggling to maintain an adequately educated nursing workforce [29]. While a few of our respondents felt the HAC policy imposed an undue burden on providers in general and nurses in particular, we were unable to identify patterns of organizational characteristics that related to their responses—the determination of other organizational drivers (*e.g.*, patient safety culture, individual belief systems, et al.) was beyond the scope of this study.

These observations are complementary to the work of Kurtzman et al., which explored the impact of the performance-based payment incentives on nursing [30]. That study focused on workforce concerns of hospital nurses at a time of significant anxiety just before the HAC policy was implemented. Certainly, the timing of the earlier work may have accounted for the difference in tone between that study and ours. It also should be noted that our sample differed significantly from that of the prior study, and that the use of qualitative methodology does not permit generalization of findings. Of note, this work identified no themes related to financial impact of non-payment. One possible explanation for the absence is that the actual financial impact on hospitals from HAC policy as it was first implemented in 2008 was determined to be very small [31]. An alternative explanation is that CNOs were more focused on the operational response and less focused on the financial impact of the HAC policy than other respondents (i.e., CEOs) might have been. Finally, our aim was not to identify the financial impact of non-payment, but to understand how the threat of non-payment influenced organizational responses in general and implementation of QI strategies in particular.

This small qualitative study has several limitations. Notably, our interviewees represented NICHE hospitals, which are atypical when compared with all American hospitals. First, NICHE hospitals have made a tangible commitment to strive for excellence in geriatric nursing. Additionally, the proportion of NICHE hospitals with Magnet designation, a designation reflecting demonstrated excellence in nursing practice, is above that of hospitals nationwide. This orientation most certainly influenced their interest in addressing the geriatric, nursing-sensitive conditions that appear on the HAC list (pressure ulcers, falls with injury and CAUTI). Second NICHE hospitals are overwhelmingly not-for-profit hospitals and our findings may not be representative of the experiences of for-profit hospitals. Finally, we interviewed chief nursing officers rather than fiscal officers, quality officers, or frontline providers. Therefore we cannot present a definitive picture of the organizational response to the HAC policy in these hospitals. Nonetheless, we used standard qualitative techniques, including several strategies to ensure methodologic rigor, to ensure that the themes derived from the interviews accurately reflected the respondents’ experiences. As is typical in qualitative research, our sample size was small, but data saturation was achieved. While the results cannot be generalized, it is likely that the themes will resonate with the experience of other hospitals.

## Conclusion

In this study of chief nursing officers at NICHE hospitals, HAC-focused QI activities were implemented in a dynamic environment. While the CMS’s HAC policy was just one of many factors influencing implementation of HAC prevention activities, it served the important role of drawing attention and resources to the targeted conditions – particularly those not previously in the spotlight. An understanding of the timing and scope of QI activities implemented in response to the HAC policy must factor in condition-specific implementation considerations that can be conceptualized with the help of the translational research paradigm. Future refinements of the HAC policy must carefully consider the many contextual factors that influence the implementation of prevention activities for each HAC.

## Competing interests

The authors declare that they have no competing interests.

## Author’s contributions

HW was responsible for the project conception, design, writing, and oversight of all phases of the project. AR participated in recruiting and interviewing participants, data analyses, and drafting the manuscript. VD led the analytic effort and participated in drafting the manuscript. EC provided input into the study design, assisted with recruiting, participated in the analytic effort, and drafting the manuscript. All authors read and approved the final manuscript.

## Funding

This work was funded by a 2009 Atlantic Philanthropies Health and Aging Policy Fellowship Award to Dr. Wald. Dr. Wald also acknowledges receipt of a Paul Beeson Career Development Award from the National Institute on Aging and the American Federation for Aging Research (K23AG034544).
